# Communication rehabilitation in sub-Saharan Africa: A workforce profile of speech and language therapists

**DOI:** 10.4102/ajod.v5i1.227

**Published:** 2016-09-09

**Authors:** Karen Wylie, Lindy McAllister, Bronwyn Davidson, Julie Marshall

**Affiliations:** 1 ENT Department, Korle Bu Teaching Hospital, Australia; 2 Faculty of Health Sciences, University of Sydney, Australia; 3 Department of Audiology & Speech Pathology, the University of Melbourne, Australia; 4 Health Professions Department, Manchester Metropolitan University, United Kingdom

## Abstract

**Background:**

There is an urgent global need to strengthen rehabilitation services for people with disabilities. In sub-Saharan Africa, rehabilitation services for people with communication disabilities continue to be underdeveloped. A first step in strengthening services for people with a communication disabilities is to understand the composition and conditions of the current workforce.

**Objectives:**

This research describes a sample of the speech and language therapists (SLTs) working in SSA (excluding South Africa). This study explores the characteristics of this workforce, including their demographics, education, experience and geographical stability.

**Method:**

A mixed-methods survey was used to collect data from SLTs within Anglophone countries of SSA. Completed surveys were received from 33 respondents working in 44 jobs across nine countries. Analysis included descriptive and non-parametric inferential statistics. This study reports on a subset of descriptive and quantitative data from the wider survey.

**Results:**

A background profile of SLTs across the region is presented. Results indicated that the workforce of SLTs comprised a mix of local and international SLTs, with university-level education. Local SLTs were educated both within and outside of Africa, with more recent graduates trained in Africa. These data reflected the local emergence of speech and language therapy training in SSA.

**Conclusion:**

This sample comprised a mix of African and international SLTs, with indications of growing localisation of the workforce. Workforce localisation offers potential advantages of linguistic diversity and stability. Challenges including workforce support and developing culturally and contextually relevant SLT practices are discussed.

## Introduction

Despite increasing global focus on rights and equity for people with disabilities (World Bank & World Health Organization 2011), rehabilitation services for people who have communication disabilities in the Majority World remain low priority (Hartley 1998; Olusanya, Ruben & Parving 2006; Wylie *et al*. 2013). [Note: the terms Majority and Minority World are used in this article to represent outdated terms ‘developing’ and ‘developed’ countries].

Communication is critical to human interaction and a basic human right (International Communication Project 2014). Communication can be affected by a diverse range of conditions which impact the ability of an individual to communicate. Communication disability has been shown to have a significant effect on participation in family life, social life, education, work and community roles. There is evidence to suggest that having a communication disability alone, without other disabilities, is associated with poorer social, educational and employment outcomes (Davidson *et al*. 2008; Johnson, Beitchman & Brownlie 2010; Law, Reilly & Snow 2013; McCormack *et al*. 2010).

Access to rehabilitation services is essential to ensure that people with communication disabilities (PWCD) have the opportunity to:

attain and maintain their maximum independence, full physical, mental, social and vocational ability, and full inclusion and participation in all aspects of life. (United Nations 2006, Article 26)

Despite this criticality, rehabilitation services for people with disabilities in sub-Saharan Africa (SSA), including communication disabilities, continue to be underdeveloped and in many places unavailable (World Bank & World Health Organization 2011).

The term ‘rehabilitation’ in this article is used to represent services including both rehabilitation and habilitation and which are:

a set of measures that assist individuals who experience, or are likely to experience, disability to achieve and maintain optimal functioning in interaction with their environments. (World Bank & World Health Organization 2011, p. 96)

This type of rehabilitation falls under the health domain of the community-based rehabilitation (CBR) matrix (World Health Organization 2010) and is often referred to as health-related rehabilitation (Nganwa, Batesaki & Mallya 2013).

Rehabilitation services operate across a range of paradigms, from specialist rehabilitation and therapy services to community-based and community-driven models of rehabilitation. The medical model of rehabilitation has dominated services in the Minority World and been imported to Africa as part of a colonial legacy (Nixon *et al*. 2015).

Simultaneously, across the Majority World, many countries, including those of SSA, have adopted the CBR model of rehabilitation (AfriCAN 2016), which uses community-based workers with minimal formal training to provide generic rehabilitation services at a community level.

Yet rehabilitation continues to fail to meet the needs of people with disabilities in the region, including PWCD. In a series of surveys, only 26% – 55% of PWD surveyed across four Southern African nations reported receiving any type of medical rehabilitation from any provider, with services received primarily being physical rehabilitation (Eide & Loeb 2006; Eide & Kamaleri 2009; Loeb & Eide 2004).

Rehabilitation is largely a human endeavour. To provide effective rehabilitation services, having the right number and mix of workers is key (Gupta, Castillo-Laborde & Landry 2011). In 2006, the World Health Organization described a clear mandate for the health workforce – ‘… get the right workers with the rights skills in the right place doing the right things!’ (p. XX). While rehabilitation is multifaceted with a scope that must extend beyond that of health systems, the same principle can extend to the rehabilitation workforce.

Who are the right workers for communication disability rehabilitation? Human resource planning is essential to ensure that appropriate rehabilitation services can be provided to those in the community who require them (World Bank and World Health Organization 2011). Understanding of the existing workforce, including their ‘social characteristics and work functions’, is needed to improve planning, policy and services (Chen *et al*. 2004, p. 1989). Yet there is little information on the composition, skill set, location and activities of the rehabilitation workforce (Gupta *et al*. 2011; WHO 2009).

In the Minority World where medical rehabilitation is commonplace, communication rehabilitation is frequently provided by speech and language therapists (SLTs). SLTs are professionals with specialist skills in rehabilitation of communication and swallowing disabilities. The modern profession emerged in the late nineteenth century in Europe and the United States. The biomedical origins and philosophy of the profession are euro-centric (western) in both origin and belief (Nixon *et al*. 2015; Pillay & Kathard 2015).

In the Majority World, with a mix of rehabilitation models in place, it is likely that the limited existing communication disability rehabilitation services available are provided by a range of people, including SLTs; therapy assistants/mid-tier workers; other professionals, such as teachers; CBR workers; traditional medicine practitioners and family members.

Research exploring the SLT workforce in SSA is timely as the profession may be growing in the region. Speech and language therapy training programmes have commenced in a number of SSA countries in recent years (Barrett & Marshall 2013; Topouzkhanian & Mijiyawa 2013) frequently supported by institutions from the Minority World.

An examination of the characteristics of the SLT workforce and the roles undertaken by SLTs will assist in our understanding of the current state of this emerging profession and how it fits in the jigsaw of services for communication disability rehabilitation in SSA. Such information has the potential to give rise to more critical discussion of how such rehabilitation services for communication disability should be organised in Africa and the relevance of the profession of SLT for this Majority World region.

This study reports on one component of research from a broad workforce survey of SLTs. It describes the demographic and educational characteristics and experience levels of a sample of the existing workforce of SLTs in SSA. This paper is a prelude to a companion paper, which reports on the nature, type and organisation of the work SLTs do in this region. Combined, the two data sets will create a preliminary exploration of this workforce providing rehabilitation services to PWCD.

### Literature Review

Historically, terms such as ‘communication disorders’ have been widely used to represent difficulties with effective communication and interaction. This term represents an impairment-focused concept. The term ‘communication disability’ is increasingly used as it recognises the complex interplay between the social and biological aspects of the person’s reality. Hartley (1998) provided a description of people with communication disability as those whose

‘…ability to communicate is affected by their response to an impairment and/or social and contextual factors which interrelate with each other and with the person themselves, resulting in impaired communication skills.’(p. 277)

Communication disabilities can be experienced by people in a multitude of ways. Communication disability can exist in isolation – as in the case of people who experience communication disability as a result of a specific communication impairment (e.g. developmental verbal dyspraxia impacting their ability to engage with the world) – or as part of another biomedical condition – such as cerebral palsy or stroke. Communication disabilities may be developmental (from birth) and experienced in different ways at different stages across a lifespan. They may be acquired as the result of trauma or disease, impacting people for part of their lives. Communication disabilities may vary in severity or impact. Others with communication ‘impairments’ may have communication difference rather than a communication disability – such as a person who stutters, or someone who is a proficient augmentative communication user. Such individuals would not be considered to have a communication disability if they are competent communicators who participate effectively in social and occupational roles. It is the lived reality of difference that determines whether someone has a communication disability.

The way rehabilitation services are offered is inextricably linked to the way disability is viewed. Early disability concepts were grounded in the medical model of disability, which saw disability as resulting from an individual’s impairments in body systems (World Health Organization 2002). In contrast, proponents of the social model of disability argued that disability is socially constructed, created by the barriers of society to the inclusion of all (Shakespeare & Watson 2002; World Health Organization 2002). Currently, the predominant disability theory is the biopsychosocial model of disability which considers that disability is created through an interplay of biological, psychological, environmental and social factors (World Health Organization 2002). With widespread adoption of the biopsychosocial model of disability through the mechanism of the International Classification of Functioning, Disability and Health (World Health Organization 2002), communication rehabilitation practices are evolving that recognise the role of both the individual and the environment in communication and interaction (Sherratt *et al*. 2011; Threats 2008).

Another important lens for thinking about communication disability rehabilitation in the Majority World is post-colonialism. This perspective is a form of critical disability studies that considers the power imbalances in relationships, influenced by ongoing impacts of the colonial legacy (Sherry 2007). The adoption of ‘western’ or euro-centric professions (Nixon *et al*. 2015), including speech and language therapy, without regard to local conceptualisations about communication disability may be considered another form of post-colonialism (Hickey *et al*. 2012). In SSA, where rehabilitation services for communication disability are few, there is a critical need to create services relevant to both culture and context, rather than replicating services, which were designed for other cultural groups (Kathard & Pillay 2013; Pillay & Kathard 2015). It is currently unclear if the profession of speech and language therapy in SSA operates in the region in the same way as in the Majority World, or if the profession, its conceptualisations about communication disability practice are evolving to meet the specific needs of the region.

Understanding rehabilitation in SSA is a complex endeavour. Two key models of ‘rehabilitation’ are utilised in the region – CBR and the medical model of rehabilitation (Haig *et al*. 2009; Nganwa *et al*. 2013; World Health Organisation 2010). The medical model is frequently associated with more specialised rehabilitation providers, who typically offer services specifically for a particular concern (i.e. visual rehabilitation, communication rehabilitation). CBR is widely embraced in the region (AfriCAN 2016). While CBR originated from a need for medical rehabilitation (World Health Organization 2010) the re-visioning of CBR has seen it evolve into a broad-based vehicle for progression of the rights, inclusion and quality of life for people with disabilities (ILO, UNESCO & WHO 2004). CBR is multisectoral and uses local resources to design community-oriented programmes across a range of dimensions, including health-related rehabilitation. There is recognition that there is a place for both more specialised rehabilitation and CBR in the struggle to produce a range of accessible and relevant services and supports for people with disabilities in the region (Nganwa *et al*. 2013) with medical rehabilitation forming a subset of the 2010 CBR guidelines (World Health Organization 2010).

Challenges in the provision of rehabilitation services are unsurprising given the difficulties faced by SSA in securing both health and rehabilitation workforces. Substantial issues continue to hamper efforts to build the health workforce in SSA, including training availability, migration of health workers, ongoing skill development and maintenance, supervision and inequitably distributed services (Anyangwe & Mtonga 2007; Touré *et al*. 2013; World Health Organization 2006). SSA represents 24% of the world’s health burden but has only 3% of global health workers and the lowest density of health workers globally (Anyangwe & Mtonga 2007; World Health Organization 2006). There is a paucity of information about the size and composition of the global rehabilitation workforce, both in medical rehabilitation and CBR (Gupta *et al*. 2011; World Bank and World Health Organization 2011), including in SSA (Haig *et al*. 2009; Olusanya *et al*. 2006; Tinney *et al*. 2007).

The allied health professions, including physiotherapy, speech and language therapy and occupational therapy, are key service providers in rehabilitation. In a global review of the health rehabilitation workforce, Gupta *et al*. (2011) found one of the lowest densities of allied health professionals globally in SSA, with many countries having less than 0.5 workers per 10 000 population.

Despite a lack of research in this field, it is acknowledged that speech and language therapy is rare or non-existent in many countries in the Majority World (World Bank and World Health Organization 2011), including SSA. In one of the few SLT workforce studies in the region, Fagan and Jacobs (2009) reported on speech and language therapy and ENT service availability across 18 SSA countries using key-informant methodology. Their data indicated an average of 0.0008 SLTs per 10 000 population in these countries, when South Africa (where SLT training has long been established) was excluded from their data. This equates to around one SLT per 12 million people. Five countries reported having no SLTs, including Democratic Republic of Congo, Malawi, Zambia, Lesotho and Ethiopia. At that time, only one nation in the survey reported offering training in speech and language therapy (South Africa). The use of informants’ to obtain information is problematic because of the reliance on the accuracy of the informants information; however, it is consistent with other anecdotal findings (J. Bampoe, personal communication, 19 February 2014; E. Shah, personal communication, 25 September 2013; Wylie *et al*. 2012) indicating the scarcity of SLTs in the region and the presence of foreign workers.

These estimates do not provide a clear picture of what SLTs are doing in the region or how communication rehabilitation services are offered, as services may be provided by others, including CBR workers and family members. However, existing figures are illustrative of the lack of specialist skills and knowledge in communication disability in the region.

In contrast to Fagan and Jacobs (2009), this research directly asked SLTs working and living in SSA, excluding South Africa, to provide detailed information about themselves. In line with Gupta *et al*. (2011), understanding more about who these professionals are, their training and their work is key to beginning to probe the current state of speech and language therapy in SSA. Such information is important evidence to consider appropriate ways forward for the development of communication disability rehabilitation services and stimulating dialogue on the best ways for the profession of SLTs to work in an African context.

Such dialogue is critical as localised programmes for speech and language therapy are now developing in the region. In 2000, speech and language therapy training commenced in Togo (Topouzkhanian & Mijiyawa 2013), followed by Uganda in 2008 (H. Barrett, personal communication, 7 September 2015), both with collaboration from Minority World countries and both institutions have been graduating SLTs. Other training programmes are known to have commenced in several other countries in SSA, a number of these in partnership with Minority World countries. Examples are given in Table 1.

**TABLE 1:** Examples of countries with recently commenced speech and language therapy training programmes.

Country	Source
Mozambique	Instituto Superior de Ciências de Saúde (2014)
Zambia	Clasp International (2015)
Kenya	Kenyatta University (2013)
Ghana	University of Health and Allied Sciences (2015)

*Source*: Authors’ own work

Data presented in this study are part of a wider survey, which aims to further our understanding of how the profession of speech and language therapy functions in the region by describing a sample of the existing workforce, including demographics, education, language and culture, work roles and continuing education.

This study begins to explore the workforce of SLT in the region, by describing a sample of SLTs working in SSA (excluding South Africa), including their demographics (age, gender, languages), education, level of experience and intended geographical stability. This research adopts an insider–outsider perspective as SLT can be considered a western or ‘euro-centric’ profession (Nixon *et al*. 2015). The research explores differences in training, experience and demographics of the workforce from this perspective.

A future companion paper will address the nature, type and organisation of the work SLTs do in this region. It is acknowledged that this small-scale research project will provide only limited data by describing a sample of the SLT workforce. However with a scarcity of both workforce information and critical discussion in the field, it is hoped that this research will further prompt consideration of the key issues and seed further workforce and service delivery research.

## Research Method and Design

A broad workforce survey was used to explore the characteristics and conditions of SLTs or those undertaking similar work, living and working in SSA, excluding South Africa. Data were collected from April 2012 to March 2013. This data set is a subset of the larger survey data, described above. The methodological description outlines the process used in the wider survey.

### Setting

The study sought to recruit SLTs living and working in the 20 Anglophone or partially Anglophone countries of SSA. Anglophone countries were selected as this grouping of nations predominantly represents those colonised by the British and are likely to have adopted similar health and educational models. These countries are listed in Appendix 1. South Africa was excluded as it was considered to have a long established history of speech therapy education (Pillay & Kathard 2015) and has a disproportionate number of SLTs when compared to other SSA countries (Fagan & Jacobs 2009). Pragmatic issues of available research funding, timeframe and language issues prohibited the inclusion of other countries in the sample.

Inclusion criteria for survey recipients included self-identifying as an SLT or similar and residence for 6 months or longer in one of the target countries.

### Materials

Because of a lack of appropriate existing survey instruments, a purpose-designed survey was developed using the process described by Punch (2003). Aims and research questions were developed by the research team, following literature review, for five key areas: workforce characteristics, education, language and culture, employment and work roles, and continuing education. Key variables and conceptual definitions were developed, variables listed and refined, research constraints identified and priority areas determined, and survey items developed and extensively reviewed by the research team who hold significant experience as SLTs in Majority World countries, including SSA. The survey was piloted with six SLTs who had previously worked in Majority World countries and modified following feedback. To improve face and content validity, pilot participants were asked to provide feedback on both survey scope and content, including the wording and sequencing of the individual items, and acceptability of time taken to complete the survey. Formal validity and reliability assessments were not within the scope of this small-scale exploratory study. The final survey contained a total of 186 items and took 45–60 minutes to complete. While this resulted in a lengthy survey, which may have negatively influenced response rates, it capitalised on the access to the participants to examine a cross-section of issues.

The survey was offered in four modalities – online (SurveyMonkey), email with attachment (Microsoft Word), paper copy or by telephone, in an attempt to meet the varying circumstances of the respondents, in contexts where mail and Internet services may be unreliable. The survey used open-ended and closed-ended questions that allowed collection of both quantitative and qualitative data. This study reports on a descriptive and quantitative data subset from the overall survey, using data from 24 items. Data reported in this study relate to 15 factual, open (text-based) items (e.g. country of residence) and 9 closed (categorical) items. Variables reported in this data subset are factual (e.g. age, qualifications) (Punch 2003).

### Sampling

Sampling the workforce of SLTs in SSA was extremely difficult as there was no sampling frame available via previous workforce studies or workforce statistics in the region. Snowball sampling (Sadler *et al*. 2010) was used for this ‘hard-to-reach’ workforce to attempt to utilise local networks existing between SLTs in the region. Snowball sampling is a commonly used sampling methodology for hard-to-reach populations where probability sampling is not possible (Handcock & Gile 2011). While recognising that this sampling methodology was unlikely to provide a representative workforce sample, the team considered this as appropriate for this exploratory research in order to begin to identify and describe workforce and service issues in the region.

This study was a small-scale exploration of workforce issues. It is acknowledged that snowball sampling offers inherent bias, including possibilities of attracting those with stronger networking or identifying those with similar educational, linguistic and cultural backgrounds (Handcock & Gile 2011). As the profession grows and systems improve, it is hoped that more representative sampling options may become feasible. This study describes the characteristics and issues of this particular workforce sample and information must be interpreted conservatively.

### Procedure

Initial contact was made with SLTs, individuals working in the disability sector, organisations in the fields of disability or health, international volunteering organisations, professional associations and academics. Initial contact was made with individuals and organisations known to the researchers or who were identified through Internet searches. Ninety initial contacts were made with individuals and organisations, and they were asked to consider forwarding the information about the survey to known SLTs in the target countries.

Contacts were provided with a link to the online survey, an MS Word version of the survey for email use, and instructions for requesting a telephone survey. Written surveys were distributed to eligible participants during the East African conference on communication disability in Kampala, Uganda, in 2012. Survey return was completion of the online survey, via email, into a completed survey return box or via telephone. Because of a lack of regional workforce data and the use of snowballing methodology, it was not possible to calculate response rates.

### Analyses

Responses to completed surveys were proofed and entered into a purpose-designed excel spreadsheet. Open responses were coded according to apriori categories (e.g. identification of African or non-African nationality) for each variable. Variables were collated into relevant groups according to research objectives and descriptive analysis undertaken, with statistical analysis performed when relevant.

Descriptive statistics were used to explore the sample. Non-parametric statistics (Mann–Whitney, Chi-square) were used for inter-group comparisons, including differences by nationality grouping and training location. Non-parametric statistics were selected because of the likely non-normal distribution of the respondents. Statistical analyses were performed using SPSS software (v22, IBM).

## Ethical considerations

Ethical approval was granted by the University of Queensland, Australia (Reference Number 2011-SOMILRE-0018). All participants received a Participant Information Sheet before commencing the survey. Return of the survey indicated informed consent. Participants were not asked to provide identifying details. Online survey data were protected by Secure Sockets Layer using both server authentication and data encryption. Electronic data were password protected and regularly backed up onto a secure server. Hardcopy data were stored in a secured area. To avoid potential identification of individual respondents because of the small numbers of SLTs present in each country, data have been aggregated where necessary.

## Results

Surveys were obtained from 33 respondents, from 9 of the 20 target countries. The lack of multiple respondents from many countries may represent either very low actual numbers of SLTs in each country or poor effectiveness of the snowball sampling methodology. Informal reports are suggestive of low numbers of SLTs in a number of target countries (Fagan & Jacobs 2009; E. Shah, personal communication, 25^th^ September, 2013). Sampling may have been more effective in countries where the research team held more established contacts (e.g. Kenya). Response modalities are outlined in Table 2.

**TABLE 2:** Survey responses by modality (*n* = 33)

Modality	Number of responses
Paper	14 (42%)
Online	12 (36%)
Email	4 (12%)
Telephone	3 (9%)

*Source*: Authors’ own work

The number of responses by country of residence is listed in Table 3. Because of the small numbers of respondents, data were clustered for analysis and presentation to ensure confidentiality.

**TABLE 3:** Survey responses by country of residence (*n* = 33).

Country	*n*	%
Ghana	5	15
Kenya	9	27
Malawi	1	3
Namibia	1	3
Nigeria	1	3
Rwanda	2	6
Tanzania	1	3
Uganda	12	36
Zimbabwe	1	3
**TOTAL**	**33**	**100**

*Source*: Authors’ own work

### Demographics: Nationality/regionality

Twenty-one respondents (64%) indicated they were resident in their home country. Of the 12 foreign respondents (36%) 1 was from another African country (*n* = 1, 8%), 10 were from a European country (*n* = 10, 83%) and 1 was from the Asia-Pacific region (*n* = 1, 8%). Further analysis clustered respondents by nationality groupings (i.e. African and non-African nationality clusters) in accordance with the regional perspective adopted in this research.

### Demographics: Age and gender

Respondents ranged in age from 25 to 59 years with a mean age of 36.0 years. A Mann–Whitney test indicated that age was not significantly different between African and non-African nationality groups [African nationality (Mdn = 37); non-African nationality (Mdn = 30) *U* = 82.5, *p* = 0.143].The majority of respondents were women (*n* = 24, 73%).

### Demographics: Languages spoken

**FIGURE 1:**
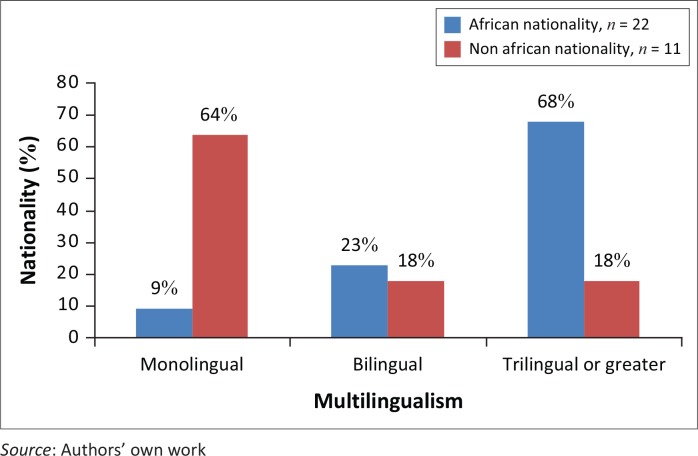
Multilingualism, by respondent nationality group.

Respondents indicated that they spoke between one and eight languages [mean of 2.9, mode 3, median 3]. A Mann–Whitney test indicated that African nationality respondents (Mdn = 3) spoke significantly more languages than their non-African peers (Mdn = 1) [*U* = 43.500, *p* = 0.002]. The distribution of multilingualism by nationality group is given in Figure 1. Unsurprisingly, a Mann–Whitney test demonstrated that African nationality respondents (Mdn = 1) spoke significantly more African languages than non-African respondents (Mdn = 0) [*U* = 36.000, *p* = 0.001]. Two non-African respondents considered themselves fluent in an African language. These respondents indicated that they had a partner or spouse from the country of residence.

### Qualifications: Speech and language therapy

Participants self-reported qualifications in speech and language therapy or fields related to clinical practice in communication disability. They also listed qualifications in unrelated fields. It was not within the scope of this project to verify qualifications.

**FIGURE 2:**
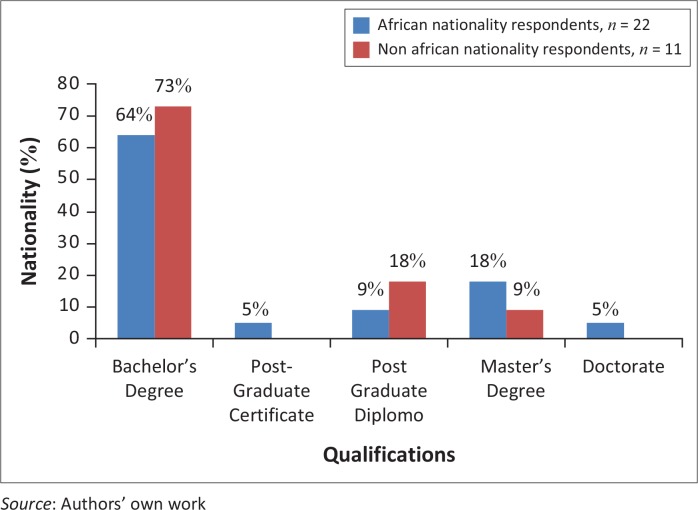
Highest qualification in speech and language therapy or related area by nationality group.

All respondents indicated holding formal qualifications in the field, ranging from Bachelor’s degree to PhD. The majority of overall respondents reported a Bachelor’s qualification (67%, *n* = 22) as their highest qualification in the field. Similar patterns of highest qualifications were evident between African and non-African nationals (Figure 2).

Five respondents (15%) indicated they had received subsequent higher level SLT-related qualifications following their initial training. Four African nationality respondents (qualifications *n* = 6) had pursued higher training in related fields both inside and outside Africa. One non-African national had received higher qualifications in their home country (qualifications: *n* = 1).

### Qualifications: Other fields

Approximately half of the respondents indicated holding additional qualifications in fields not specifically related to speech and language therapy (52%, *n* = 17). There was no significant difference between African and non-African nationality groups in self-reported other qualifications [χ²(1) = 1.52, *p* = 0.218]. The type of ‘other’ qualifications reported ranged from health and education (e.g. occupational therapy, teaching) to unrelated disciplines (e.g. law, management).

### Training: Region of entry-level speech and language therapist training

More than half of the African nationality respondents had received their first (entry-level) speech and language therapy qualification inside Africa (*n* = 13, 59%). The majority of this subgroup (*n* = 12) had trained in Uganda. Of those who trained in Uganda, 10 were Ugandan nationals, with the remaining 2 from other East African nations.

Of the 41% (*n* = 9) of African nationality respondents who had received their entry-level SLT qualification outside Africa, the majority had trained in the United Kingdom, with others reporting training in the United States, Russia and Canada. All non-African nationality respondents reported training in Minority World countries (100%, *n* = 11) (e.g. the United Kingdom, United States and the Netherlands). These data are outlined in Table 4.

**TABLE 4:** Region of entry-level speech and language therapy training.

Nationality Grouping	Trained in SSA	Trained outside SSA	Total
**African nationality respondents**	13 (59%)	9 (41%)	22 (100%)
**Non-African nationality respondents**	0 (0%)	11 (100%)	11 (100%)
**All respondents**	13 (39%)	20 (61%)	33 (100%)

*Source*: Authors’ own work

The age of African nationals who trained outside Africa (Mdn = 37) was not significantly different to those who trained on the continent (Mdn = 38) [Mann–Whitney *U* = 46.5, *p* = 0.697].

African nationality respondents who trained outside Africa (Mdn = 8) had been SLTs for longer than the non-African nationals trained in the region, as they reported significantly greater number of years since completion of their training than their non-African peers (Mdn = 3), [Mann–Whitney *U* = 9.000, *p* = 0.001].

### Experience: Working with people with communication disabilities

**FIGURE 3:**
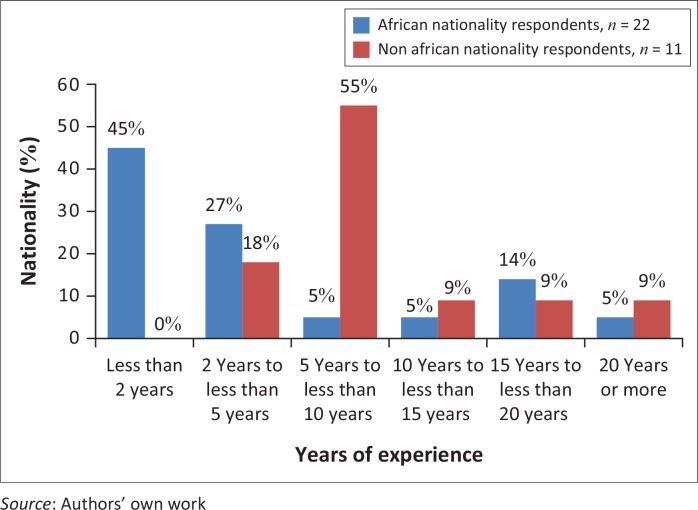
Self-reported years of experience working with people with communication disabilities, by nationality group.

Participants were asked to indicate a range of years of experience they had in working with PWCD. Experience levels were clustered at the less experienced end of the range, with over half (*n* = 18, 54%) of all respondents reporting less than 5 years of experience. When examined by nationality group, experience levels of African nationality respondents were negatively skewed, with 45% (*n* = 10) of respondents reporting less than 2 years of experience. Experience levels of non-African nationals were primarily clustered between 2 and 10 years (*n* = 8, 73%). Self-reported number of years of experience working with PWCD is outlined in Figure 3.

Non-African nationals self-reported significantly more experience working with PWCD than their African peers [Mann–Whitney *U* = 58.50, *p* = 0.015].

African nationality respondents who trained outside of Africa also self-reported significantly more experience working with PWCD than their African nationality peers who trained inside Africa [Mann–Whitney *U* = 26.500, *p* = 0.042].

### Stability: Intention to stay

**FIGURE 4:**
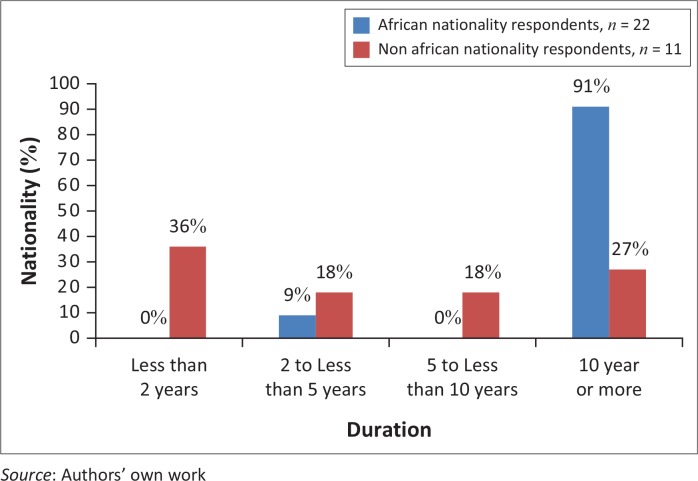
Intended duration of residence, by nationality group.

As an indicator of geographical stability, respondents were asked to estimate how long they were likely to remain in their current country of residence. African nationality respondents reported strong intention to stay, with 91% (*n* = 21) of respondents indicating an intention to remain in country permanently. In contrast, there was a spread of intended length of residence for non-African nationality respondents (Figure 4). Over one-third of non-African respondents (36%, *n* = 4) reported an intended residence of less than 2 years.

### Stability: Reason for residence

**FIGURE 5:**
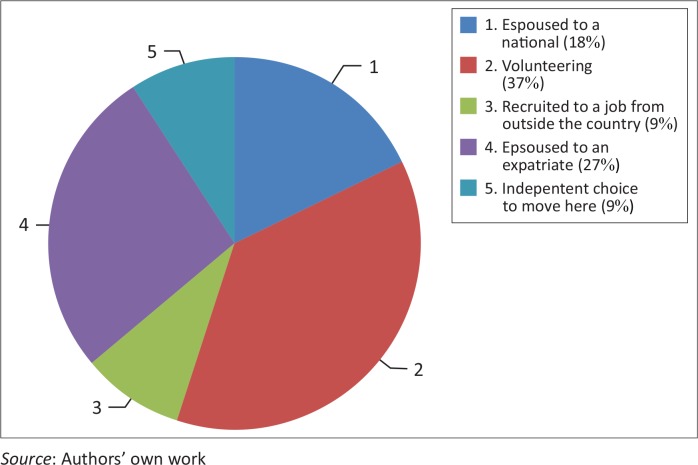
Reason for residence, Non-African nationalities, *n* = 11.

The small sample of non-African nationality respondents were asked to indicate their main reason for being a resident in Africa (Figure 5). One third (*n* = 4, 36%) reported coming on volunteer postings. Other reasons for residence included spouses of expatriate workers (*n* = 3, 27%), espoused to a national of the country (*n* = 2, 18%), recruited to a particular job (*n* = 1, 9%) and arriving independently and remaining in the country (*n* = 1, 9%).

Respondents who intended to stay for shorter durations (less than 2 years) had a variety of motivations for residence, including volunteering (*n* = 2), recruited to a job from outside Africa (*n* = 1) and a spouse who is an expatriate worker (*n* = 1). Respondents who had a partner or spouse from the residence country indicated strong intention to stay – from 10 years to permanently.

### Validity

This research explores demographic and educational profiles of a sample of SLTs. These data address a range of key issues of interest, but do not make claim to be representative of the population of SLTs in the African continent, particularly with the likely rapidly changing workforce. Sampling issues outlined impacted on sample size and may have impacted internal validity. The survey was developed by the researchers with significant expertise in the field. Positive feedback regarding relevance of the content was received from pilot participants, who had experience of working in the African context.

## Discussion

### Outline of the results

This study describes the workforce characteristics of a sample of 33 SLTs working in 9 countries of Anglophone SSA and explores the demographics, training, clinical experience and geographical stability of this sample.

While the amount of data is small, it provides useful insight into the SLT workforce emerging in the region.

Participants were distributed across age groups from their 20s to their 40s with fewer respondents aged 50 or over. Women comprised 75% of this sample. Gender imbalance in the speech and language therapy profession is consistently noted in the Minority World countries, with a low proportion of men in the workforce [e.g. 2.5% Australia (Health Workforce Australia 2014) and 3.7% the United States (American Association of Speech and Hearing 2013)].

SLTs indicated speaking a range of languages, although African SLTs were significantly more multilingual and spoke more African languages than their non-African peers. This is particularly relevant because of the polyglot nature of African communities and the need to provide intervention in mother tongue where possible.

An important feature of this sample was the mix of foreign and local workers, with one third of respondents originating from countries outside the region. Despite the small study numbers, this sample concurs with anecdotal reports (J. Bampoe, personal communication, 19 February 2014; E. Shah, personal communication, 25 September 2013), suggesting that foreign nationals makeup a substantial proportion of the small speech and language therapy workforce in the region.

All respondents held formal university-level qualifications for practice as a SLT. Qualifications reported were similar to entry-level qualification for speech and language therapy in the Minority World, where entry qualifications vary from Bachelor’s to entry-level coursework Master’s degrees (RCSLT n.d.; Speech Pathology Australia 2005).

Training location varied, with African nationals reporting training both within SSA and outside, while all non-African nationality respondents trained outside of the region. African nationals who trained outside the continent had been qualified for longer than those who had trained within Africa. This pattern is suggestive of a shift – from training outside the region to training inside the region, as training options develop. The small standard deviation in years since training completion for the African trained group lends credibility to the data, as training programmes in SSA have only recently commenced (i.e. in the past decade) and many of the African respondents who qualified in Africa had completed their training in Uganda, which commenced in 2008 (H. Barrett, personal communication 7^th^ September 2015).

Qualifications of speech and language therapy in this sample mirror education and SLT qualifications seen in the Minority World. This is unsurprising given the partnerships seen in establishing training programmes for SLT in SSA described in the introduction.

Experience with working with PWCD varied across the sample, with the foreign workforce reporting significantly more years of experience than their local peers, and a wide range of experience levels reported among African nationality SLTs. African graduates trained outside the continent reported more experience than African nationals trained within the continent. This may represent the shift in the availability of training to more recent African-based training. While there was a relatively even age distribution of respondents, almost three quarters of African nationality respondents indicated that they had less than 5 years’ experience of working with PWCD, indicating a likely late entry into the field. This may be because of the relatively recent commencement of SLT training in Uganda, where a large proportion of respondents trained.

If SLT is to play a part in the myriad of services for communication disability, then ensuring workforce stability is key (Gupta *et al*. 2011). Data from this sample suggest that an African SLT workforce may ultimately offer the continent more stability than the use of non-African SLTs. Non-African nationality respondents indicated a rapid intended turnover, with over half (54%) indicating that they would remain in country for less than 5 years. Such rapid geographical mobility compromises workforce stability and may present challenges to service continuity, depending on the type of work that shorter term SLTs undertake.

This rapid turnover of non-African nationality SLTs is particularly important in light of the differing linguistic, cultural and disability service contexts in which these foreigners operate. SLTs from outside may require substantial training and support in an endeavour to provide culturally relevant practice (Hickey *et al*. 2012) and time in country may contribute to such cultural relevance.

While a great deal is written about volunteering and volunteerism, foreigners in this sample reported a range of reasons for being in Africa. While one third of foreign respondents came as volunteers, others were present for a variety of reasons. Further research on their motivations and experiences as SLTs in SSA and the perspectives of the African stakeholders on the contributions and stability offered by foreigners would add value to the literature on volunteering.

### Implications

There is now increasing recognition that both health-related rehabilitation and CBR have a place in the spectrum of rehabilitation services. There has been widespread adoption of CBR in the region (AfriCAN 2016), but there continues to be a key role for health-related rehabilitation, as it underpins other elements of the CBR process (Nganwa *et al*. 2013).

The profession of SLT is historically and conceptually euro-centric (Nixon *et al*. 2015; Pillay & Kathard 2015) and is commonplace in contexts where CBR is not widespread. The expansion of SLT in SSA prompts further debate about its relevance and future. Is the profession of speech and language therapy truly part of the mix of ‘right workers’ for communication disability rehabilitation in the region? If so, how can this profession with its roots in the medical model evolve responsively to African culture and contexts? This research does not answer these questions but consideration of workforce characteristics, stability and education of SLT should prompt discussion about the future and relevance of the profession.

If SLT is to be part of an African response to communication disability rehabilitation, growth of African training is critical. Apart from the obvious advantages of language in direct service provision, SLTs originating from and embedded within a culture will be ultimately best placed to reconceptualise the communication rehabilitation appropriately.

Barrett and Marshall (2013) stress the importance of:

sustainable, culturally appropriate, nuanced, and accessible services for PWCD, and this can only be achieved when local professionals are empowered to develop services in their own communities. (p. 50)

Relevant and competent rehabilitation is providing a service that is respectful of, and responsive to, an individual’s values, beliefs, preferences and language and is well integrated with the local health environment and systems (Hickey *et al*. 2012; Laleman *et al*. 2007).

The presence of foreign workers in the mix of SLTs in the region presents both diverse challenges and opportunities for SLT in the provision of relevant and responsive rehabilitation services. Use of a foreign (volunteering) workforce in other domains has been shown to add value in ways that include knowledge transfer, service development, capacity building and influx of resources (Laleman *et al*. 2007). Conversely, the use of foreign workforces may have disadvantages including their limited cultural awareness and language skills, imposition of ideas and dependence (Hickey *et al*. 2012; Laleman *et al*. 2007). In the case of SLT, post-colonialism may include the imposition of rehabilitation practices which may not be best suited to the culture and context. It is critical for all stakeholders to continue to be mindful of the subtle power and cultural dimensions that impact rehabilitation services.

While many African nations have European languages as their national language, following a legacy of colonialism, African communities are typically polyglot.

Cultural and linguistic challenges have been specifically identified as limiting the effectiveness of ‘outsiders’ in the volunteer health service literature (Laleman *et al*. 2007). Cultural and linguistic competencies are an important component of effective service provision, particularly in a field that has language and communication at its core. Yet foreigners in this study show rapid turnover and limited linguistic diversity. Increasing availability of SLTs who have appropriately diverse language skills and cultural backgrounds should assist in the development of appropriate communication rehabilitation practices in Africa, including the development of culturally and linguistically appropriate research and resources (Topouzkhanian & Mijiyawa 2013).

The relatively recent advent of speech and language therapy training in Uganda appears to have bolstered the profession of SLTs in this region, with just over a third of respondents to this survey having completed their training in Uganda (*n* = 12, 36%). Ultimately, the practices adopted by SLTs may depend on factors such as where and how they are trained and the influences apparent in the training. For example, SLTs trained in the United States may receive more clinical specialist training, whereas SLTs trained in bespoke training programmes in Africa may potentially receive more focus on local service contexts, training others, CBR and public health interventions (Barrett & Marshall 2013; Wickenden *et al*. 2001; Wylie *et al*. 2014). Where and how training is established and whether local culture is central to SLT training may shape the evolution of SLT practices in SSA. SLTs with extensive experience in the region are well placed to assist this evolution. Further research in this field in curriculum and practice in the region is required.

To produce an effective workforce, it is well recognised that after qualification, workers need ongoing systems for support and to have accessible continuing education (Chen *et al*. 2004; World Health Organization 2006). For practicing SLTs, access to ongoing support specific to the culture and context is required (Rochus, Lees & Marshall 2014). Individuals well placed to offer such support include the African SLTs with substantial experience in the region. However, the demand for support from these relative few SLTs may be disproportionate to availability. Using foreign SLTs with regional experience and cultural understanding, typical of the non-African nationality respondents in the survey, may be useful in providing support to the growing profession (Rochus *et al*. 2014). With rapidly emerging mobile technologies, support could be provided either locally or remotely (see Nuffield Foundation 2015). Explicitly identifying professional support needs at this juncture is critical as graduates enter the workforce to ensure their work becomes both high quality and relevant to the culture and context.

## Limitations of the study

The results cannot be generalised to the population of SLTs, as a non-probability sampling method was used and response rates cannot be determined. Integrity of the results is dependent on how accurately respondents have interpreted and responded to survey items. Selection bias was likely, because of the use of snowball sampling. Potential respondents may have been more likely to show interest in participation if they had previous contact with the research team, rather than via unsolicited invitation. Despite these limitations, this study offers preliminary data and insights into issues that are emerging as the SLT workforce grows and develops in SSA.

## Conclusion

This research described a sample of the small SLT workforce in the region and identified a mix of both local and foreign workers in the provision of SLT services. SLTs were predominantly women, consistent with patterns in the Majority World. African nationals reported higher rates of multilingualism and were likely to be less geographically transient than their foreign peers. Qualifications in the sample of SLTs mirrored qualifications to practice as an SLT in the Majority World.

African nationals had less experience working with PWCD than the foreigners in the sample; however, there was a wide range of experience among African nationals. African nationals who trained outside the region had more experience than their locally trained peers supporting reports of growth in regional speech and language therapy training.

This small-scale research project provides a profile of the characteristics, education and experience and stability of a sample of the SLT workforce in SSA. The growth of the local SLT workforce offers potential advantages in linguistic competence and increased workforce stability. Growth in the profession also gives rise to questions about the relevance of SLT in the region, how it fits with existing rehabilitation models and how practices derived in a European belief framework can best evolve to meet the needs of the African populations they serve.

This small-scale research is an attempt to further understanding of one element of the workforce for communication disability rehabilitation in SSA. Improving our understanding of the rehabilitation workforce will allow a more strategic approach to workforce and service development (Gupta *et al*. 2011; Touré *et al*. 2013).

## Appendix 1

### Countries included in the study: Anglophone or partially Anglophone countries of Sub-Saharan Africa

1. Botswana (Republic of Botswana)
2. Gambia (Republic of The Gambia)
3. Ghana (Republic of Ghana)
4. Kenya (Republic of Kenya)
5. Lesotho (Kingdom of Lesotho)
6. Liberia (Republic of Liberia)
7. Malawi (Republic of Malawi)
8. Namibia (Republic of Namibia)
9. Nigeria (Federal Republic of Nigeria)
10. Sierra Leone (Republic of Sierra Leone)
11. Swaziland (Kingdom of Swaziland)
12. Tanzania (United Republic of Tanzania)
13. Uganda (Republic of Uganda)
14. Zambia (Republic of Zambia)
15. Zimbabwe (Republic of Zimbabwe)
16. Eritrea (State of Eritrea)
17. South Sudan (Republic of South Sudan)
18. Sudan (Republic of Sudan)
19. Cameroon (Republic of Cameroon)
20. Rwanda (Republic of Rwanda)

## References

[CIT0001] AfriCAN, 2016, *CBR directory for Africa*, viewed 22 January 2016, from http://afri-can.org/cbr-directory-for-africa/

[CIT0002] American Association of Speech and Hearing, 2013, *Highlights and trends: Member and affiliate counts, year-end 2013*, viewed 15 June 2015, from http://www.asha.org/uploadedFiles/2013-Member-Counts-Year-End-Highlights.pdf

[CIT0003] Anyangwe S.C. & Mtonga C, 2007, ‘Inequities in the global health workforce: The greatest impediment to health in sub-Saharan Africa’, *International Journal of Environmental Research and Public Health* 4(2), 93–100. http://dx.doi.org/10.3390/ijerph2007040002 17617671 10.3390/ijerph2007040002 PMC3728573

[CIT0004] Barrett H. & Marshall J, 2013, ‘Implementation of the World Report on Disability: Developing human resource capacity to meet the needs of people with communication disability in Uganda’, *International Journal of Speech-Language Pathology* 15(1), 48–52. http://dx.doi.org/10.3109/17549507.2012.743035 23190008 10.3109/17549507.2012.743035

[CIT0005] Chen L., Evans T., Anand S., Boufford J.I., Brown H., Chowdhury M. et al, 2004, ‘Human resources for health: Overcoming the crisis’, *The Lancet* 364(9449), 1984–1990. http://dx.doi.org/10.1016/S0140-6736(04)17482-5 10.1016/S0140-6736(04)17482-5 15567015

[CIT0006] Clasp International, 2015, *SLP master’s program*, viewed 10 September 2015, from http://claspinternational.org/programs/slp-masters-program.

[CIT0007] Davidson B., Howe T., Worrall L., Hickson L. & Togher L, 2008, ‘Social participation for older people with aphasia: The impact of communication disability on friendships’, *Topics in Stroke Rehabilitation* 15(4), 325–340. http://dx.doi.org/10.1310/tsr1504-325 18782736 10.1310/tsr1504-325

[CIT0008] Eide A. & Loeb M, 2006, *Living conditions among people with activity limitations in Zambia: A national representative study*, SINTEF, Oslo.

[CIT0009] Eide A.H. & Kamaleri Y, 2009, *Living conditions among people with disabilities in Mozambique: A national representative study*, SINTEF, Oslo.

[CIT0010] Fagan J.J. & Jacobs M, 2009, ‘Survey of ENT services in Africa: Need for a comprehensive intervention’, *Global Health Action* 2, 1–7. http://dx.doi.org/10.3402/gha.v2i0.1932 10.3402/gha.v2i0.1932 PMC2779942 20027268

[CIT0011] Gupta N., Castillo-Laborde C. & Landry M.D, 2011, ‘Health-related rehabilitation services: Assessing the global supply of and need for human resources’, *BMC Health Services Research* 11(1), 276–287. http://dx.doi.org/10.1186/1472-6963-11-276 22004560 10.1186/1472-6963-11-276 PMC3207892

[CIT0012] Haig A.J., Im J., Adewole A., Nelson V.S. & Krabek B, 2009, ‘The practice of physical medicine and rehabilitation in sub-Saharan Africa and Antarctica: A white paper or a black mark?’, *Disability & Rehabilitation* 31(13), 1031–1037. http://dx.doi.org/10.1080/09638280902803765 19802923 10.1080/09638280902803765

[CIT0013] Handcock M.S. & Gile K.J, 2011, ‘Comment: On the concept of snowball sampling’, *Sociological Methodology* 41(1), 367–371. http://dx.doi.org/10.1111/j.1467-9531.2011.01243.x 10.1111/j.1467-9531.2011.01243.x PMC8797839 35095124

[CIT0014] Hartley S, 1998, ‘Service development to meet the needs of ‘people with communication disabilities’ in developing countries’, *Disability & Rehabilitation* 20(8), 277–284. http://dx.doi.org/10.3109/09638289809166083 9651686 10.3109/09638289809166083

[CIT0015] Health Workforce Australia, 2014, *Health workforce Australia 2014: Australia’s health workforce series – speech pathologists in focus*, Health Workforce Australia, Canberra, viewed 1 August 2015, from https://www.hwa.gov.au/resources/publications

[CIT0016] Hickey E.M., Archibald C., McKenna M. & Woods C, 2012, ‘Ethical concerns in voluntourism in speech-language pathology & audiology’, *Perspectives in Global Issues in Communication Sciences & Related Disorders* 2, 40–48. http://dx.doi.org/10.1044/gics2.2.40

[CIT0017] ILO, UNESCO & WHO, 2004, *CBR: A strategy for rehabilitation, equalization of opportunities, poverty reduction and social inclusion of people with disabilities*, Joint position paper, ILO, Geneva.

[CIT0018] Instituto Superior de Ciências de Saúde, 2014, *Therapia da fala*, viewed 10 September 2015, from http://www.iscisa.ac.mz/db2/cursos/graduacao/terapia/terapia-da-fala.html

[CIT0019] International Communication Project, 2014, *The universal declaration of communication rights*, viewed 14 September 2015, from http://www.communication2014.com/wp-content/uploads/2014/09/English-Declaration.pdf

[CIT0020] Johnson C.J., Beitchman J.H. & Brownlie E, 2010, ‘Twenty-year follow-up of children with and without speech-language impairments: Family, educational, occupational, and quality of life outcomes’, *American Journal of Speech-Language Pathology* 19(1), 51–65. http://dx.doi.org/10.1044/1058-0360(2009/08-0083) 19644128 10.1044/1058-0360(2009/08-0083)

[CIT0021] Kathard H. & Pillay M, 2013, ‘Promoting change through political consciousness: A South African speech-language pathology response to the World Report on Disability’, *International Journal of Speech-Language Pathology* 15, 84–89. http://dx.doi.org/10.3109/17549507.2012.757803 23323822 10.3109/17549507.2012.757803

[CIT0022] Kenyatta University, 2013, *Special needs Education*, viewed 10 September 2015, from http://www.ku.ac.ke/schools/education/index.php/departments/special-needs-education

[CIT0023] Laleman G., Kegels G., Marchal B., Van Der Roost D., Bogaert I. & Van Damme W, 2007, ‘The contribution of international health volunteers to the health workforce in sub-Saharan Africa’, *Human Resources for Health* 5(1), 19–19. http://dx.doi.org/10.1186/1478-4491-5-19 17672889 10.1186/1478-4491-5-19 PMC1971668

[CIT0024] Law J., Reilly S. & Snow P.C, 2013, ‘Child speech, language and communication need re-examined in a public health context: A new direction for the speech and language therapy profession’, *International Journal of Language and Communication Disorders* 48(5), 486–496. http://dx.doi.org/10.1111/1460-6984.12027 24033648 10.1111/1460-6984.12027

[CIT0025] Loeb M.E. & Eide A.H, 2004, *Living conditions among people with activity limitations in Malawi: A national representative study*, SINTEF, Oslo.

[CIT0026] McCormack J., McLeod S., Harrison L.J. & McAllister L, 2010, ‘The impact of speech impairment in early childhood: Investigating parents’ and speech-language pathologists’ perspectives using the ICF-CY’, *Journal of Communication Disorders* 43(5), 378–396. http://dx.doi.org/10.1016/j.jcomdis.2010.04.009 20510422 10.1016/j.jcomdis.2010.04.009

[CIT0027] Nganwa A.B., Batesaki B. & Mallya J.A, 2013, ‘The link between health-related rehabilitation and CBR’, in Musoke G. & Geiser P. (eds.), *Linking CBR, Disability and Rehabilitaton*, 59–71, CBR Africa Network, Bangalore.

[CIT0028] Nixon S.A., Cockburn L., Acheinegeh R., Bradley K., Cameron D., Mue P.N. et al, 2015, ‘Using postcolonial perspectives to consider rehabilitation with children with disabilities: The Bamenda-Toronto dialogue’, *Disability and the Global South* 2, 570–589.

[CIT0029] Nuffield Foundation, 2015, *Services for communication disability*, viewed 12 September 2015, from http://www.nuffieldfoundation.org/services-people-communication-disabilities-uganda

[CIT0030] Olusanya B.O., Ruben R.J. & Parving A, 2006, ‘Reducing the burden of communication disorders in the developing world: An opportunity for the millennium development project’, *Journal of the American Medical Association* 296, 441–444. http://dx.doi.org/10.1001/jama.296.4.441 16868302 10.1001/jama.296.4.441

[CIT0031] Pillay M. & Kathard H, 2015, ‘Decolonizing health professionals’ education: Audiology & speech therapy in South Africa’, *African Journal of Rhetoric* 7, 193–227.

[CIT0032] Punch K, 2003, *Survey research: The basics*, Sage, London.

[CIT0033] Rochus D., Lees J. & Marshall J, 2014, ‘Give me someone who has been there: Reflections on the experience of mentoring SLTs in East Africa’, *The Bulletin* 746, 12–14.

[CIT0034] Royal College of Speech and Language Therapy, n.d., *A career in speech and language therapy*, RCSLT, London.

[CIT0035] Sadler G.R., Lee H., Lim R.S. & Fullerton J, 2010, ‘Recruitment of hard-to-reach population subgroups via adaptations of the snowball sampling strategy’, *Nursing & Health Sciences* 12(3), 369–374. http://dx.doi.org/doi:10.1111/j.1442-2018.2010.00541.x 20727089 10.1111/j.1442-2018.2010.00541.x PMC3222300

[CIT0036] Shakespeare T. & Watson N, 2002, ‘The social model of disability: An outdated ideology?’, *Research in Social Science and Disability* 2, 9–28. http://dx.doi.org/10.1016/S1479-3547(01)80018-X

[CIT0037] Sherratt S., Worrall L., Pearson C., Howe T., Hersh D. & Davidson B, 2011, ‘‘Well it has to be language-related’: Speech-language pathologists’ goals for people with aphasia and their families’, *International Journal of Speech-Language Pathology* 13, 317–328. http://dx.doi.org/10.3109/17549507.2011.584632 21793777 10.3109/17549507.2011.584632

[CIT0038] Sherry M, 2007, ‘(Post)colonising disability’, *Wagadu* 4, 10–22.

[CIT0039] Speech Pathology Australia, 2005, *Dual entry to the speech pathology profession*, SPA, Melbourne viewed 14 January 2016, from http://www.speechpathologyaustralia.org.au/library/position_statements/Dual_Entry_to_the_profession.pdf

[CIT0040] Threats T.T, 2008, ‘Use of the ICF for clinical practice in speech-language pathology’, *International Journal of Speech-Language Pathology* 10, 50–60. http://dx.doi.org/10.1080/14417040701768693

[CIT0041] Tinney M.J., Chiodo A., Haig A. & Wiredu E, 2007, ‘Medical rehabilitation in Ghana’, *Disability & Rehabilitation* 29(11–12), 921–927. http://dx.doi.org/10.1080/09638280701240482 17577726 10.1080/09638280701240482

[CIT0042] Topouzkhanian S. & Mijiyawa M, 2013, ‘A French-speaking speech-language pathology program in West Africa: Transfer of training between Minority and Majority World countries’, *International Journal of Speech Language Pathology* 15(1), 58–64. http://dx.doi.org/10.3109/17549507.2012.757802 23323818 10.3109/17549507.2012.757802

[CIT0043] Touré B., Atchénémou Avocksouma D., Nyoniii J. & Ahmatii A, 2013, ‘Road map for scaling up human resources for health for improved health service delivery in the African Region 2012–2025’, *African Health Monitor* 18 viewed 28 January 2016, from https://www.aho.afro.who.int/en/ahm/issue/18/reports/road-map-scaling-human-resources-health-improved-health-service-delivery

[CIT0044] United Nations, 2006, ‘Convention on the rights of persons with disabilities’, viewed 2 January 2016, from http://www.un.org/disabilities/convention/conventionfull.shtml 10.1515/9783110208856.203 18348362

[CIT0045] University of Health and Allied Sciences, 2015, *Departments*, viewed 10 September 2015, from http://www.uhas.edu.gh/index.php/allied-health-sciences/departments-of-alliedhealth

[CIT0046] Wickenden M., Hartley S., Kodikara S., Mars M., Sell D., Sirimana T. et al, 2001, ‘Collaborative development of a new course and service in Sri Lanka’, *International Journal of Language & Communication Disorders* 36(s1), 315–320. http://dx.doi.org/10.3109/13682820109177904 11340804 10.3109/13682820109177904

[CIT0047] World Bank and World Health Organization, 2011, *World Report on Disability*, World Health Organization, Geneva.

[CIT0048] World Health Organization, 2002, *Towards a Common Language for Functioning, Disability and Health: ICF* The International Classification of Functioning, Disability and Health (Vol. WHO/EIP/GPE/CAS/01.3). WHO, Geneva.

[CIT0049] World Health Organization, 2006, *The World Health Report 2006 – Working Together for Health*, World Health Organization, Geneva.

[CIT0050] World Health Organization, 2009, *Monitoring human resources for health-related rehabilitation services.: Vol. 7. Spotlight on health workforce statistics*, World Health Organization, Geneva Viewed 3 August 2016, from http://www.who.int/hrh/statistics/spotlight_7_en.pdf

[CIT0051] World Health Organization, 2010, *Community based rehabilitation: CBR guidelines*, World Health Organization, Geneva. 26290927

[CIT0052] Wylie K., McAllister L., Davidson B. & Marshall J, 2013, ‘Changing practice: Implications of the World Report on Disability for responding to communication disability in under-served populations’, *International Journal of Speech-Language Pathology* 15(1), 1–13. http://dx.doi.org/10.3109/17549507.2012.745164 23323813 10.3109/17549507.2012.745164

[CIT0053] Wylie K., McAllister L., Davidson B., Marshall J. & Law J, 2014, ‘Adopting public health approaches to communication disability: Challenges for the education of speech-language pathologists’, *Folia Phoniatrica et Logopaedica* 66(4–5), 164–175. 25790923 10.1159/000365752

[CIT0054] Wylie K., McAllister L., Davidson B., Marshall J. & Wickenden M, 2012, ‘Overview of issues and needs for new SLP university programs in developing countries’, paper presented at the East African Conference on Communication Disability, Kampala, 12–15 January.

